# VITILIGO TREATMENT WITH VITAMINS, MINERALS AND POLYPHENOL SUPPLEMENTATION

**DOI:** 10.4103/0019-5154.57613

**Published:** 2009

**Authors:** Akrem Jalel, Gaigi Siala Soumaya, Mohamed Hédi Hamdaoui

**Affiliations:** 1 *From the Unité de Recherche sur les Composés Antioxydants, Stress Oxydant, Eléments Traces et Maladies Métaboliques, Ecole Supérieure des Sciences et Techniques de la Santé de Tunis*; 2 *From the Service d'anatomie-pathologie et d'Embryologie Departement de Foetopathologie, CHU-Centre de Maternité et de Néonatologie de Tunis.*

**Keywords:** *Polyphenol*, *vitiligo*, *vitamins*

## Abstract

**Background:**

Mammalian pigmentation results from the synthesis and accumulation of photo protective epidermal melanin. Melanin was formed from the amino acid precursor L-tyrosine within specialized cells, the melanocytes. Oxidative stress has been suggested to be the initial pathogenetic event in melanocyte degeneration with H_2_O_2_ accumulation in the epidermis of patients with active disease. Auto immunity has been also suggested as another hypothesis in the pathogenesis of depigmentation disorders. Topical corticosteroids and phototherapy as common treatment modalities have been prescribed in patients with vitiligo. However, they are often not effective and safe (epidermal atrophy). Therefore, research for alternative therapies continues.

**Aims:**

To evaluate the beneficial effects of a supplementation with antioxidant vitamins (A, C, E) and minerals (zinc, selenium) for vitiligo treatment.

**Methods:**

Forty experimental autoimmune vitiligo mice C57BL6, aged from 5 to 12 months showing visible signs of induced vitiligo, were sequentially randomized into five parallel groups (8 mice per group). Each group mice was allocated an identical pre coded cage. the first group (SZV) received the ED + 1,4 g zinc (Zn) + 0.04 g selenium (Se) + vitamins (A 118 UI, C 8,5 mg, E 5,4 UI) /kg diet, the second group (PSZV) received the ED + 1,4 g zinc (Zn) + 0.04 g selenium (Se) + vitamins (A 118 UI, C 8.5 mg, E 5,4 UI)/kg diet + Polyphenol orally, the group 3 (PSZ) received the ED + green tea decoction prepared from 100 g/l (polyphenol orally) + 1,4 g Zn + 0.04 g Se, the 4 (P) received the ED + green tea decoction prepared green tea decoction prepared from 100 g/l, the control group 5(C) received the ED + distilled water. Cure was defined as repigmentation of treated sites. Photographic and optical techniques were used both at the baseline and on weekly basis.

**Results:**

By the end of the study, mices showed visible repigmentation. Using the investigator's global assessment, therapeutic success in terms of a clear repigmentation documented in 70% of treated mice.

**Conclusion:**

Our findings suggest that an antioxidant supplementation is significantly beneficial in contributing superior clinical efficacy to cure vitiligo.

## Introduction

Vitiligo equally affects men and women and can occur at any age. The etiology of the disease is still unknown.[1] Besides the most popular autoimmune theory, several groups have recently shown the involvement of oxidative stress in the pathophysiology of this disease.[2,3] The mechanism by which oral psoralens and UV-A radiation (PUVA) stimulate melanocyte proliferation in vitiligo and other hypopigmentary diseases is not known.[4] PUVA is immunosuppressive as a result of its effect on cytotoxic T lymphocytes. This action of PUVA on T lymphocytes could also be the explanation for the therapeutic effect of PUVA on vitiligo. Any theory of therapy must explain not only the action on cytotoxic T lymphocytes, but also the stimulation of melanocyte proliferation and the migration of melanocytes to the epidermis from the hair follicle.[5] *In vivo* exposure of human epidermis to solar-stimulated UV-irradiation causes changes in the enzymatic antioxidant defence system which, in turn, is accompanied by increased levels of oxidative stress.[6] Photosensitizing agents are also known to generate reactive oxygen species.[7] These problems may be prevented by the use of antioxidants.

## Materials and Methods

### Animals and diets

Forty experimental autoimmune vitiligo mices C57BL weighing between 20 and 35 g, aged from 5 to 7 months and showing visible signs of induced vitiligo, were sequentially randomized into five parallel groups. Each group mice was allocated an identical pre coded cage. Upon arrival, mice were fed a commercial semi-synthetic diet for one week to adapt them in our laboratory conditions and equilibrate their initial weights. The semi-synthetic diet is composed per g/100 g dry weight: Carbohydrates: 51; Proteins: 21.8; lipids: 5.7; linoleic acid 1.5; L-methionine: 0.4; L-cysteine: 0.6; Choline 0.2; vitamin mixture: 1.7 and mineral mixture: 3.2. This diet contained 0.190 g FeSO_4_. 7 H_2_O, corresponding to 40 mg iron/kg diet which is roughly similar to the iron content of experimental diet (ED) figured in Table 1. Once the initial weights were equilibrated, the mice were randomly assigned into five groups of eight animals each. They were housed individually in stainless-steel wire cages. Room temperature was maintained at 25 ± 2°C, and lit daily from 7 am to 7 pm. During the experimental period (22 weeks), the mice were given the ED ad libitum with or without green tea decoction as follows:, the first group (SZV) received the ED + 1,4 g zinc (Zn) + 0.04 g selenium (Se) + vitamins (A 118 UI, C 8,5 mg, E 5,4 UI) /kg diet, the second group (PSZV) received the ED + 1,4 g zinc (Zn) + 0.04 g selenium (Se) + vitamins (A 118 UI, C 8,5 mg, E 5,4 UI) /kg diet + Polyphenol orally, the group 3 (PSZ) received the ED + green tea decoction prepared from 100 g/l (polyphenol orally) + 1,4 g Zn + 0.04 g Se, the 4(P) received the ED + green tea decoction prepared green tea decoction prepared from 100 g/l, the control group 5(C) received the ED + distilled water. Food intake was recorded throughout the experimental period and quantified by weighing the amount of food spilled and refused. The daily supplement (vitamins + Zn + Se) consumed was deduced from the intake diet by calculation. The volume of tea decoction consumed by each mouse was measured daily. Before tea intake, each mouse was given about 7 ml of ultra pure water to prevent dehydration. At the end of the experimental period, mice were weighed and then killed by decapitation. Blood was drawn in vacutainer tubes, centrifuged at 3000 rpm for 10 min, and then the plasma was removed and frozen for the analysis of plasmatic parameters.

**Table 1:** Composition of the experimental diet

Ingredients	Amounts g/kg diet
Powder skim	400
Milk*	55
Vegetable oil	299
Maize starch	180
Sucrose	1
L-cysteine	1.5
Choline	0.2
FeSO_4_†	20
CaCO_3_	20
Na_3_PO_4_	20
Na Cl	5
K Cl	5
Al_2_ (SO_4_)_3_.18 H_2_O‡	1.2
Vitamin mixture§	10
Mineral mixture‖	2.5

* As source of protein (Inesfood, Tunisia) provides 140 g proteins/kg.

† Amount of FeSO_4_ added to the ED provides 40 mg iron/kg which is similar to that of the semi-synthetic diet. Protein and iron widely covered the requirements of rats.

‡ Aluminum sulfate (1.2 g of Al_2_ (SO_4_)3.18 H_2_O), corresponds to 100 mg pure aluminum, was mixed in the maize starch of diets of groups 2, 4, 5 and 6.

§ Vitamin mixture (per Kilogram diet); synthetic vitamin A concentrate = 10,000 IU; cholecalciferol = 2,000 IU; α-tocopherol acetate = 4 mg; pyridoxine hydrochloride = 4 mg; ribofavin sodium phosphate = 3 mg; nicotinamide = 20 mg; ascorbic acid = 100 mg; dexpanthenol = 8 mg, and finely powdered sucrose to make10 g.

‖ Mineral mixture (grams per kilogram diet): MgSO_4_.7H_2_O = 1.85; ZnSO_4_. 7H_2_O = 0.50; MnSO_4_. 4H_2_O = 0.15; CuSO_4_. 5H_2_O = 0.020; KIO_3_ = 0.0015; FeSO_4_. 7H_2_O = 0.200; Na_2_SeO_3_ = 0.022.

### Preparation of the ED

The ED was prepared by mixing ingredients and chemical compounds listed in Table 1 in a stainless blender. A no toxicity dose level of Al sulfate (1.2 g of Al_2_ (SO_4_)_3_.18 H_2_O), corresponds to 100 mg pure Al/kg diet was mixed with the maize starch of the ED. This amount of Al is comparable to that present in green tea decoction prepared from 100 g/l. We noted that the oral lethal dose of Al sulfate in mice and rats ranged from 200 to 1000 mg of Al per kg of body weight. The mineral and the vitamin supplements of the experimental diet were prepared as recommended by the APRIA.[2] The homogeneous diet was transformed into a piece of cake, then dried at 45°C and stored at 4°C for a short time.

### Preparation of tea decoction

Tea decoction was freshly prepared throughout the experimental period. Amounts of 25, 50 or 100 g green tea leaves (*Camellia sinensis*) were soaked in hot water and boiled in 1,000 ml of ultra pure water for 20 min in a Pyrex glass (Duran), then cooled to room temperature before distribution.

### Evaluation of the treatment and antioxidant system

The mice were evaluated after three and six months of treatment. Total body photographs were taken for each mouse, including photographs of the front and back sides of mice in a standard pose. The results were scored as bad (no improvement or improvement less than 25%), moderate (improvement between 25% and 74%), and good (75% or more).

### Ethics

The local ethics committee approved the study.

### Statistics

Fisher's exact test, Mann-Whitney U test, Wilcoxon matched pairs test, and Kruskall-Wallis test were used in statistical analysis.

## Results

By the end of the study, 70% of mice showed visible repigmentation in the supplemented group. Using the investigator's global assessment, therapeutic success in terms of a clear repigmentation was documented in 72% of SZV group treated vitamins, zinc and selenium supplemented diet. Seven (87.5%) mice in the first group had good scores, (12.5%) had moderate scores. 5 mice (62.5%) in the second PSZ had good scores, two (25%) patients had moderate scores, and one (12.5%) had bad scores. Three mice (37.5%) in the second PSZV had good scores, 3 (37.5%) patients had moderate scores, and 2 (25%) had bad scores. one (12.5%) mice in the fourth group (P) had good scores, and 7 had (87.5%) bad scores. All mice (100%) in the fifth (C) group had bad scores.

## Discussion

Reactive oxygen species are capable of bleaching constitutional melanin and causing membrane lysis through lipid peroxidation reactions.[2] The use of antioxidants inhibits these effects of UV-irradiation, and antioxidants can be used as a tool for improvement of psoralen photochemotherapy.[8] The skin consists of three layers: The epidermis (outer layer), dermis (mid-layer) and hypodermis (inner layer). The green tea polyphenols aren't absorbed beyond the epidermis, so any benefits are limited to that outer layer of skin. However, the benefits seem to be significant.[9] Cells in the epidermis or keratinocytes are in a constant state of renewal. The newly formed cells, stem cells, are undifferentiated but rapidly dividing. As they push through the epidermis, they begin differentiating. During this migration and differentiation process, the cells are very active, expending and consuming vast amounts of energy.[10] In vitiligo patients, the epidermal levels of ubiquinol, vitamin E, reduced glutathione (GSH), and CAT activity were reduced. An imbalance of the intracellular redox status and a significant depletion of enzymatic and non-enzymatic antioxidants are seen in the epidermis of vitiligo patients, and represent the fingerprint of an abnormal oxidative stress leading to epidermal cell injury.[11] In classic of PUVA treatment leads to depletion of GSH levels in the skin.[12] A decreased GSH is associated with strong decreases in tyrosine hydroxylase activity and melanin production in the skin.[13] Both α-tocopherol and GSH-deficiency potentiate the susceptibility of the cells to oxidative membrane injury.[14] However, the blood levels of vitamin A,C E, super oxide dismutase, GSH, GSH peroxidase, lipoperoxides and polyunsaturated fatty acids of phospholipids in vitiligo patients are not significantly different from those of healthy age matched controls.[15] In our study, repigmentation in the mice in the first group showed a significantly greater increase than did those of the control group. The basis of this process is the antioxidante reaction of vitamins and polyphenols with unsaturated lipids and the impairment of barrier functions of biomembranes.[8] This epidermal oxidative stress may be the cause of premature melanocyte cell death. In contrast, epidermal oxidative stress in the mice in the second group (PSZV) changed only in five mice (62.5%) versus seven in the first group (SZV: 87.5%) which may be explained by the interference in their intestinal absorption between vitamin C and polyphenols. Our hypothesis is that the oxidative stress caused by the photochemical reaction in these vitiliginous mice was inhibited by vitamin A, C, E as an antioxidant in addition to polyphenol, zinc and selenium which are enzymes cofactors: Glutathione peroxidase, catalase which are melanocyte antioxidant defence keys. Nutritional deficiencies, both in animals and in humans, are known to alter melanin pigmentation. Copper and zinc deficiencies have been reported to induce hypopigmentation in various animals. Hypopigmentation of the skin and hair results from copper deficiency in humans; the depigmentation associated with chronic excessive molybdenum intake is related to a decreased storage of copper in the liver. Copper would seem to be of prime importance because tyrosinase is a known copper-requiring enzyme.

An imbalance in the antioxidant system and free radical-mediated damage are initial pathogenetic events in melanocyte degeneration in vitiligo.[3] Therefore, to achieve manifest repigmentation, antioxidants may be kept in mind as an adjuvant in the treatment of vitiligo patients. Vitamin E may prevent an oxidative distress resulting from PUVA therapy, and may improve the number of good responses to PUVA, although this improvement is not significant statistically and clinically in vitiligo mices in our study.

## Conclusion

Our findings suggest that an antioxidant supplementation is significantly beneficial in contributing superior clinical efficacy to cure vitiligo.
